# Decreased peripheral basophil counts in urticaria and mouse model of oxazolone-induced hypersensitivity, the latter suggesting basopenia reflecting migration to skin

**DOI:** 10.3389/fimmu.2022.1014924

**Published:** 2022-09-29

**Authors:** Izumi Kishimoto, Ni Ma, Riko Takimoto-Ito, Chisa Nakashima, Atsushi Otsuka, Andrew F. Walls, Hideaki Tanizaki, Naotomo Kambe

**Affiliations:** ^1^ Department of Dermatology, Kansai Medical University, Hirakata, Japan; ^2^ Department of Dermatology, Kyoto University Graduate School of Medicine, Kyoto, Japan; ^3^ Department of Dermatology, Kindai University Graduate School of Medical Sciences, Sayama, Japan; ^4^ Immunopharmacology Group, Clinical and Experimental Sciences, University of Southampton, Southampton, United Kingdom

**Keywords:** Basophils, Urticaria, Basopenia, *in vitro* IgE, ovarian response, Oxazolone

## Abstract

A decrease in the number of basophils in the peripheral blood, or basopenia, has been noted, reflecting the activity of chronic spontaneous urticaria (CSU). Infiltration of basophils into the skin has also been reported, but the mechanism of basopenia in CSU has not been clarified. The phenomenon of basopenia during the active phase of urticaria was confirmed, and basophil numbers increased following symptom improvement in 15 out of 17 patients treated with omalizumab and in 13 of 15 patients treated with antihistamines. Our examination by immunostaining also revealed basophil infiltration of the CSU lesions, as in previous reports, but since most of our patients were already taking oral steroids, it was not considered appropriate to examine the relationship between basophil numbers in tissue and peripheral blood. Then, we used mouse model of contact hypersensitivity with a single application of oxazolone, which is known to stimulate basophil infiltration, and investigated basophil counts in the skin, peripheral blood, and bone marrow. In this model, a decrease in peripheral blood basophil numbers was observed one day after challenge, but not after 2 days, reflecting supplementation from the bone marrow. Indeed, when cultured basophils expressing GFP were transplanted into the peripheral blood, GFP-positive basophil numbers in the peripheral blood remained low even after 2 days of challenge. Despite differences among species and models, these results suggest that one reason for the decrease of basophils in the peripheral blood in CSU may involve migration of circulating basophils into the skin.

## Introduction

Basophils are the least numerous types of granulocytes in the peripheral blood (PB), generally representing less than 1% of leukocytes. They differentiate from bone marrow (BM) and enter the circulation. Basophils have basophilic granules in their cytoplasm, high-affinity IgE receptors on their surface and as is the case with mast cells, release chemical mediators such as histamine (1–3). In mice, a member of the mouse mast cell protease (mMCP) family termed mMCP-8 (with coding gene *Mcpt8*) is unique to basophils and has served as a highly specific differentiation marker for this cell type (4, 5). However, studies of the roles of basophils have been neglected in immunological research due to their presence in relatively small numbers, and they have sometimes been confused with mast cells, which reside in tissues (6).

A decrease in the number of basophils in the PB, or basopenia, has long been reported in urticaria (7). Some reports suggested that basophils in urticaria patients are impaired in IgE-mediated histamine release, but this study reported that the peripheral basophil counts in the patients with chronic spontaneous urticaria (CSU) were slightly, but not significantly, lower than in healthy subjects (8). A study in 2008 (9) examined whether the presence of autoantibodies in CSU affects the impaired histamine release from basophils, but this study did not focus on the number of basophils. In another report, however, Oliver et al. (10) evaluated observations at two time points and showed that leukocyte histamine levels, reflecting the number and presence of basophils in the PB, vary inversely with skin rash and itch scores. A systematic search of 73 CSU studies reported in 2021 (11) did not list basophil count as a predictor of the efficacy with treatment, while it has been reported that PB basophil counts are inversely correlated with CSU activity, and that antihistamine treatment increases the number of basophils in the PB (10, 12). In particular, omalizumab, a monoclonal anti-IgE antibody, was shown to be effective in the treatment of CSU (13), and in the course of validating the efficacy of omalizumab, basopenia came to the attention again (14–16), along with various functional abnormalities of basophils shown by urticaria patients (17, 18).

In addition, infiltration of basophils has been reported in the skin tissue of CSU (19–21), but its relationship to basopenia has not been investigated. Thus, we have not had direct evidence that decreasing the number of circulating peripheral basophils reflected basophil migration into tissues. In this study, we confirmed that the PB basophil count, which was decreased during the active phase of CSU, increased with successful treatment with omalizumab and antihistamines. We also confirmed that basophils were present at the lesion site of CSU by immunostaining. However, we could not directly verify whether the basopenia reflected local basophil infiltration. Therefore, the relationship between changes in basophil counts in PB and infiltration of basophils into local skin tissues with inflammation was examined using an oxazolone (OX)-induced contact hypersensitivity model, in which basophils are known to migrate to lesion sites, due to the lack of an appropriate mouse model for CSU.

## Materials and methods

### CSU and peripheral basophil counts

The patients suffering from CSU were recruited in Kansai Medical University Hospital (Hirakata, Japan). PB samples were collected before and after treatment with antihistamines or omalizumab (300 mg, every 4 weeks), along with other laboratory tests. The severity of urticaria was evaluated by urticaria activity score over 7 days (UAS7) at the outpatient examination. Patients were reevaluated 4-8 weeks after the start of omalizumab use and those who had symptom resolution or USA7 improvement were collected. For patients treated with antihistamines, blood was also collected when symptoms relieved, but the timing varied from 1 to 9 weeks, depending on the case. The basophil counts in PB were calculated from leukocyte counts and leukocyte fractions in the clinical laboratory at the hospital, and in some cases were confirmed to be CD3-/CRTH2+/CD203c+ cells by flow cytometry (FACS) using the Allergenicity Kit (Beckman Coulter, Brea, CA). Total IgE levels were also measured by electro-chemiluminescence immunoassay in the hospital’s clinical laboratory.

All human materials were approved by the Institutional Review Board of Kansai Medical University (2018199) and the study was conducted in accordance with the Declaration of Helsinki.

### Mice

C57BL/6JJmsSlc mice were purchased from Shimizu Laboratory Supplies (Kyoto, Japan). *Mcpt8^GFP^* mice on the C57BL/6J background (22) were kindly provided by Drs. Miyake and Karasuyama (Tokyo Medical and Dental University). All mice were maintained under specific pathogen-free conditions in the animal facilities with the guidelines of Kansai Medical University for animal care, and all animal studies were approved by the Institution Annimal Care and Use Committee of Kansai Medical University (22–046, 22–047).

### BM-derived cultured basophils

BM-derived cultured basophils (BMBa) were prepared as described previously (23). Briefly, BMBa were prepared by culturing BM cells in the presence of 0.3 ng/mL recombinant murine IL-3 (BioLegend, San Diego, CA) in RPMI1640 with 10% FCS for 1 week.

### OX-induced contact hypersensitivity model

OX (4-Ethoxymethylene-2-phenyl-2-oxazolin-5-one, Sigma-Aldrich, St. Louis, MO) was dissolved in ethanol. Female C57BL/6J mice at the age of 8 weeks old were percutaneously sensitized with 100 μL of 3% OX on their shaved back skin and challenged 5 days later with topical applications of either 30 μL of 1% OX on both the dorsal and ventral surfaces of their ears for challenge. Ear thickness of sedated mice was measured with a caliper (PEACOCK Dial Thickness Gauge 0.01mm type G, Ozaki MFG, Tokyo, Japan).

In some experiments, 1x10^6^ BMBa were intravenously injected through the tail vein one day before OX-challenge.

### FACS analysis

The ear skin was treated with 200 μL of Liberase solution by mixture of 10 mg of Liberase I (Roche, Basel, Switzerland) dissolve in 26 mL of RPMI-1640 medium with 1% FCS at 37°C for 1 hour, then added 20 μL of 0.5 M EDTA and incubate at 37°C for 5 min with shaking to stop the collagenase reaction. BM was harvested from the one side of femur and pressed out with 1 mL of PBS. In PB, flow-count fluorospheres (Beckman Coulter) were added to each 100 µL of samples. Then, cells from BM and PB was lysed in lysis buffer (BD Biosciences, Franklin Lakes, NJ). Single cell suspensions were obtained by FACS buffer (PBS containing 2% FCS, 0.1% sodium azide, and 1 mM EDTA) from the treated skin, BM and PB. All the samples were stained with an indicated combination of monoclonal antibody (mAb) for 30 min and analyzed by FACS Canto II (BD Biosciences). Following antibodies for flow cytometry were all purchased from BioLegend: PE/Cy7-conjugated CD45 (30F11); PE-conjugated CD49b (DX5); Pacific blue-conjugated CD117 (c-kit, 2B8); and APC-conjugated CD200R3 (Ba13). Dead cells were excluded by staining with propidium iodide (PI, Immunostep, Salamanca, Spain) or 7-AAD (BD Biosciences). Cells were analysed with FlowJo (BD Biosciences). Each cell lineages were defined in the CD45+ hematopoietic lineage cells as follows: basophils (c-kit–/CD49b+/CD200R3+), and mast cells (c-kit+/CD200R3+). The number of basophils in PB was identified by calculating the total number of CD45+ cells from the number of flow-count fluorospheres, and skin and BM basophils were accessed as a percentage of live cells evaluated for PI or 7-AAD negative.

### Histopathological analysis

Formalin-fixed, paraffin-embedded human skin samples, biopsied from the urticaria lesion, were stained with hematoxylin and eosin (HE) or a human basophil-specific mAb, BB1 (20), and mast cell tryptase-specific mAb, G3 (24), in combination with the alkaline phosphatase (AP)-conjugated secondary antibody (Vector Labs, Burlingame, CA) and color was developed with Fast-Red substrate, followed by hematoxylin counterstaining. Digital images of each slide were acquired by NanoZoomer 2.0 HT (Hamamatsu, Shizuoka) and the number of cells was counted by the related image viewing software NDP view 2 by selecting 3 parts of areas randomly at 100 µm view.

Mouse ear specimens were fixed with 4% paraformaldehyde and embedded in paraffin, and sections were stained with HE or with a basophil-specific anti-mMCP-8 (TUG8, BioLegend) with donkey anti-rat IgG (Alexa Fluor 594-conjugated, Thermo Fisher, Waltham, MA) and anti-GFP (B-2, Abcam, Cambridge, UK) with donkey anti-goat IgG (Alexa Fluor 488-conjugated, Abcam).

### Quantitative PCR

Total RNA was extracted from tissues or isolated cells by RNeasy Mini Kit (QIAGEN, Germantown, MD), followed by cDNA synthesis with SuperScript III First-Strand Synthesis System (Thermo Fisher). Q-PCR of the cDNA was performed with a Fast SYBR Green Master Mix (Thermo Fisher) by using following primer sets: Mm_Mcpt8_1_SG QuantiTect Primer Assay (Qiagen, GeneGlobe Id: QT00131565) and GAPDH as housekeeping gene: 5’-CATCACTGCCACCCAGAAGACTG and 5’-ATGCCAGTGAGCTTCCCGTTCAG. Relative expression value of *Mcpt8* was calculated by 2ΔCT for the housekeeping gene.

### Statistical analysis

Statistical differences were determined by the statistical tests stated in each figure legend using GraphPad Prism (San Diego, CA). P < 0.05 was considered statistically significant.

## Results

### Peripheral basophil count in CSU was recovered after treatment

**Figure 1:**
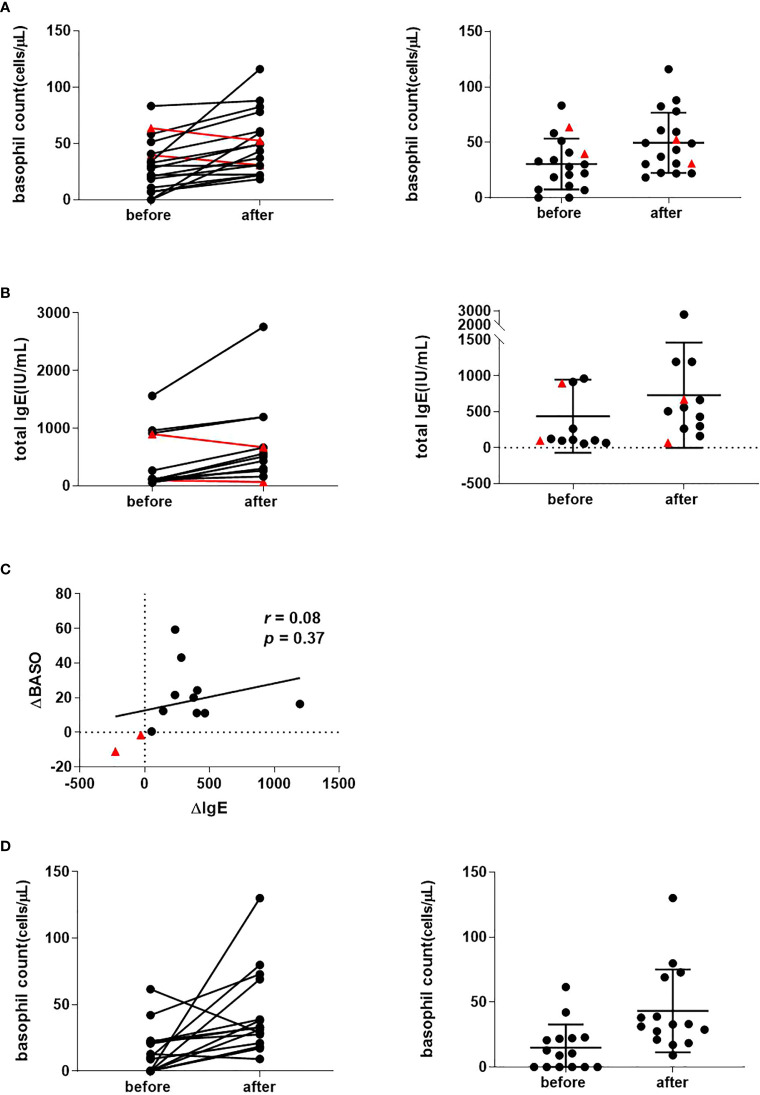
Peripheral basophil counts in 17 CSU patients before and following recovery with omalizumab treatment. **(A)** Peripheral basophil counts before and after omalizumab treatment. Left panel: Changes before and after treatment in individual cases. Right panel: Mean values before and after treatment. Data show individual values as means and SD. **(B)** Serum IgE levels before and after omalizumab treatment (n = 13). Left panel: Changes before and after treatment in individual cases. Right panel: Mean value before and after treatment. Data show individual values as mean and SD. **(C)** Correlation between changes in peripheral blood basophils and serum IgE before and after treatment (n = 13). ΔBASO = (after – before) basophil count, ΔIgE = (after – before) total IgE levels. Red triangles show a decrease in basophils after symptom improvement compared to pre-treatment. **(D)** Peripheral basophil count of CSU patients was recovered with antihistamine treatment. **(A)** Peripheral basophil count before and after antihistamine treatment (n=15) combination with corticosteroid (n = 3) and cyclosporine (n = 1). left panel: Changes before and after treatment in individual cases. right panel: Average value before and after treatment. Data show individual values as means and SD.

Of the 17 CSU patients recruited who were treated with omalizumab, we observed 15 cases in which there was a recovery in basophil counts (mean 30.4/µL rising to 50.0/µL, *p* = 0.003) (**Figure 1A**) that was accompanied with an improvement in rash as assessed by UAS7.

Successful omalizumab treatment is associated with neutralization of serum IgE and increased serum IgE levels. In patients whose serum IgE levels could be evaluated before and after treatment with omalizumab (n = 13), 11 patients had an increase in serum IgE levels as the skin rash improved (mean 437/µL rising to 730/µL, *p* = 0.014) (**Figure 1B**). There was no association between pre- and post-treatment IgE levels (*r* = 0.08, *p* = 0.37). Two cases with no increase in basophil numbers after initiation of omalizumab (indicated by red triangles in **Figure 1A** and **1B**), had no increase in IgE levels (**Figure 1C** with red triangles). When an increase in serum IgE levels was used as an indicator that omalizumab was sufficiently neutralizing IgE, the PB basophil counts increased associated with improvement in the skin rash.

Of the 15 retrospectively collected CSU patients who had been treated with antihistamines, three received concomitant oral corticosteroids and one cyclosporine. We compared the basophil count in PB before treatment and at the time the rash disappeared or was relieved by antihistamine treatment. Of the 15 cases, we observed that basophil numbers increased in 13 cases and decreased in two cases. Mean basophil counts of 14.9/µL rose to 43.1/µL after the treatment (*p* = 0.012) (**Figure 1D**).

### Basophil detection in the affected skin of CSU patients

**Figure 2:**
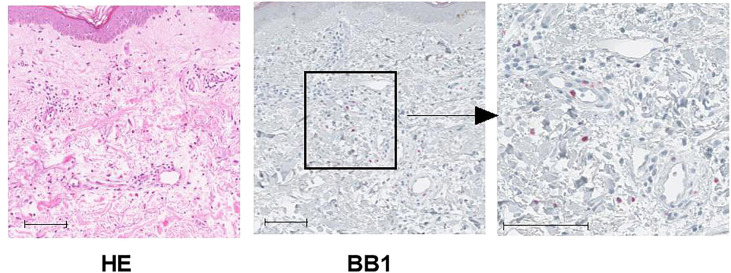
Representative image of histological findings of biopsy in urticaria lesions. This tissue is from case 4 in **Table 1**. Left panel: HE stains, Middle panel: Immunochemical stain with BB1, anti-human basophil specific mAb, scale bar = 100 µm. Right panel: Higher magnification for BB1 stain, scale bar = 100 µm.

Although skin biopsies were not usually performed in CSU cases, some retrospectively collected antihistamine-refractory cases underwent skin biopsy to differentiate them from other conditions such as collagen diseases or vasculitis. Immunostaining of biopsied tissue with basophil-specific antibody, BB1, showed basophil infiltration in the lesioned skin (**Figure 2**). As shown in **Table 1**, of the 22 CSU patients, we identified basophils in 12 samples. The number of basophils averaged 2.4 ± 5.4 in the field of observation, which was approximately 1/10 of the number of mast cells (24.5 ± 11.9) identified as tryptase-positive cells in the same field. In the present study, there was no trend toward a decrease in the number of basophils in the PB in patients with skin infiltration of basophils (**Table 1**). However, most of the patients who had skin biopsies performed were resistant to antihistamine treatment, and most of them were receiving oral steroids at the time they were referred to our hospital for skin biopsies, so we did not consider it appropriate to examine the correlation between the number of PB basophils and the number of basophils infiltrating the tissues.

**Table 1:** Mast cell and basophil counts in urticarial skin lesions and blood basophil counts.

**Case**	**G3^†^**	**BB1** ^‡^	**Blood Basophil** ^§^	**Treatment** ^¶^
1*	28	0	0	AH
2	26	3	30.9	mPSL 125 mg + BMZ 0.25 mg + AH
3*	47	0	0	AH
4	51	26	10.6	AH
5*	31	7	9.9	none
6	12	2	38.8	BMZ 0.5 mg + AH
7	20	0	0	BMZ 0.75 mg + AH
8	34	0	13.2	PSL 5 mg + AH
9	6	0	10.2	AH
10**	12	2	40	AH
11	17	0	19.8	none
12*	29	2	26.8	none
13	8	0	6.9	AH
14	15	3	50.4	AH
15	20	1	0	PSL 20 mg + AH
16	46	0	7.8	AH
17	24	0	9.7	BMZ 0.75 mg + AH
18*	26	2	14.8	PSL 10 mg + AH
19	27	1	0	PSL 10 mg + AH
20	28	0	15	mPSL 125 mg + AH
21	15	1	0	PSL 8 mg + AH
22	18	2	11.4	PSL 10 mg + AH
**Mean ± SD**	24.5 ± 11.9	2.4 ± 5.4	14.4 ± 14.2	

^†^Tryptase+ mast cells in the CSU lesion skin (/field).

^‡^Basophils in the CSU lesion skin (/field).

^§^Basophil counts in the peripheral blood (/μL).

^¶^Treatment of urticaria at the time of biopsy. Those who had received any oral corticosteroid or salazosulfapyridine, which could affect the basophil count in the peripheral blood, were marked with *, and those whose treatment before biopsy was unknown were marked with ** after the case number.

AH, antihistamine; BMZ, betamethasone; mPSL, methylprednisolone; PSL, prednisolone.

### Basophil changes in tissue and blood in mice OX model

A decrease in the number of basophils in PB may reflect migration of basophils into the tissues, but direct evidence of such migration is difficult to obtain in humans. Therefore, we decided to investigate the relationship between the decrease in basophil count in PB and basophil migration to the skin, using a mouse contact dermatitis model induced by OX, which is known to cause basophil migration to the local skin lesions (25).

**Figure 3:**
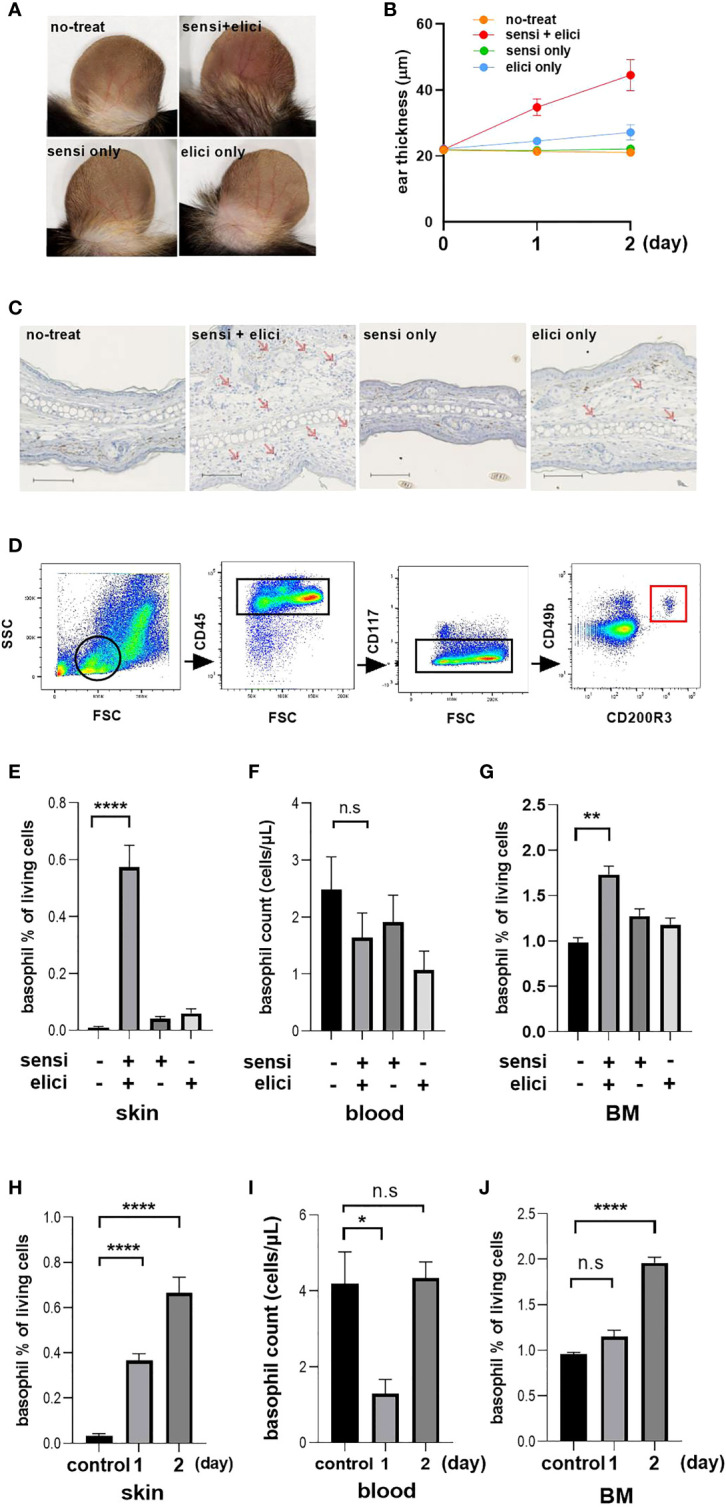
Basophil changes in tissue and blood in mouse OX model. Each experiment was conducted at least 3 times individually (each group, n = 3), and data from one of these experiments are shown as representative. **(A)** Representative image of photo taken two days after OX challenge. **(B)** Thickness of the ear. **(C)** Representative image of immunohistochemical staining of mouse ear tissue with TUG8, anti-basophil specific mAb that recognizes mMCP-8, scale bar = 100 µm. **(D)** Gating for the identification of activated basophil. From the lymphocyte and granulocyte population gated by FSC and SSC, we identified activated basophils as CD45-positive and CD117-negative and CD49b-positive and CD200R3-positive. **(E–G)** Basophil counts in skin, blood and BM two days after sensitization and challenge. **(H-J)** Basophil count at 1 day after sensitization. Controls were mice without either sensitization or challenge. *p < 0.05, **p < 0.001, ****p < 0.0001 ns. no significance. “sensi” is sensitization and “elicit” is elicitation.

Redness of the ears was observed only in the groups both treated with sensitization with 3% OX on their back skin and challenged 5 days later with 1% OX on both the dorsal and ventral surfaces of their ears (**Figure 3A**). Swelling of the ear reflecting inflammation was similarly observed only in the sensitized and elicited groups, and swelling became noticeable the day after the challenge, and was further enhanced two days later (**Figure 3B**). Two days after challenge, mice were sacrificed and skin samples were taken for immunostaining, and a large number of mMCP8-positive basophils were observed infiltrating the local skin area in the sensitized and challenged groups (**Figure 3C**), reflecting the swelling of the skin.

We further identified basophils using FACS analysis, by first gating lymphocyte fractions with forward side scatter (FSC) and side scatter (SSC), then narrowing down to single cells with basophils identified as CD45+/CD117-/CD49b+/CD200R3+ cells (**Figure 3D**). The results confirmed the presence of large numbers of basophils in the tissue of the sensitized and challenged groups, consistent with the results of immunostaining (**Figure 3E**). However, when basophils in PB were examined at this time, there was no decrease in numbers of basophils in PB similar to that in human CSU patients. Although there was a trend for a slight decrease in the number of basophils in the group challenged with OX, this did not reach significance (**Figure 3F**). Interestingly, at this time we found that the BM basophil counts were increased only in the sensitized and challenged group (**Figure 3G**).

Based on these results, we hypothesized that failure to observe a decrease in basophil numbers in the PB at 2 days post-challenge, reflecting migration of basophils to the skin at the site of inflammation, was a result of mobilization of new basophils from BM into the PB. Therefore, we decided to reexamine the basophil kinetics over time, although the skin swelling was still slight at 1 day after sensitization (**Figure 3B**). The number of infiltrating cells increased on the second day from that on the first day, reflecting the swelling of the ear (**Figure 3H**). On the other hand, when the basophil count in the PB was examined, a decrease in the PB basophil count was observed on one day after OX challenge, but it recovered to the original level two days later (**Figure 3I**). When the basophil count in the BM of the mice was examined at this time, an increase in the basophil count in the BM was observed two days after OX challenge (**Figure 3J**).

### Restoration of peripheral basophil numbers two days after challenge reflects mobilization from BM

Based on the results of the mouse OX model, it was suggested that reduction in the number of basophils in the PB one day after challenge is due to migration to the skin, but two days after challenge, the number of basophils in the PB is restored due to the mobilization of new basophils from BM. To evaluate the supplementation of basophils from BM to PB, we generated BMBa from *Mcpt8^GFP^* mice (22), which express GFP specifically in basophils, and transferred these cells into PB.

**Figure 4:**
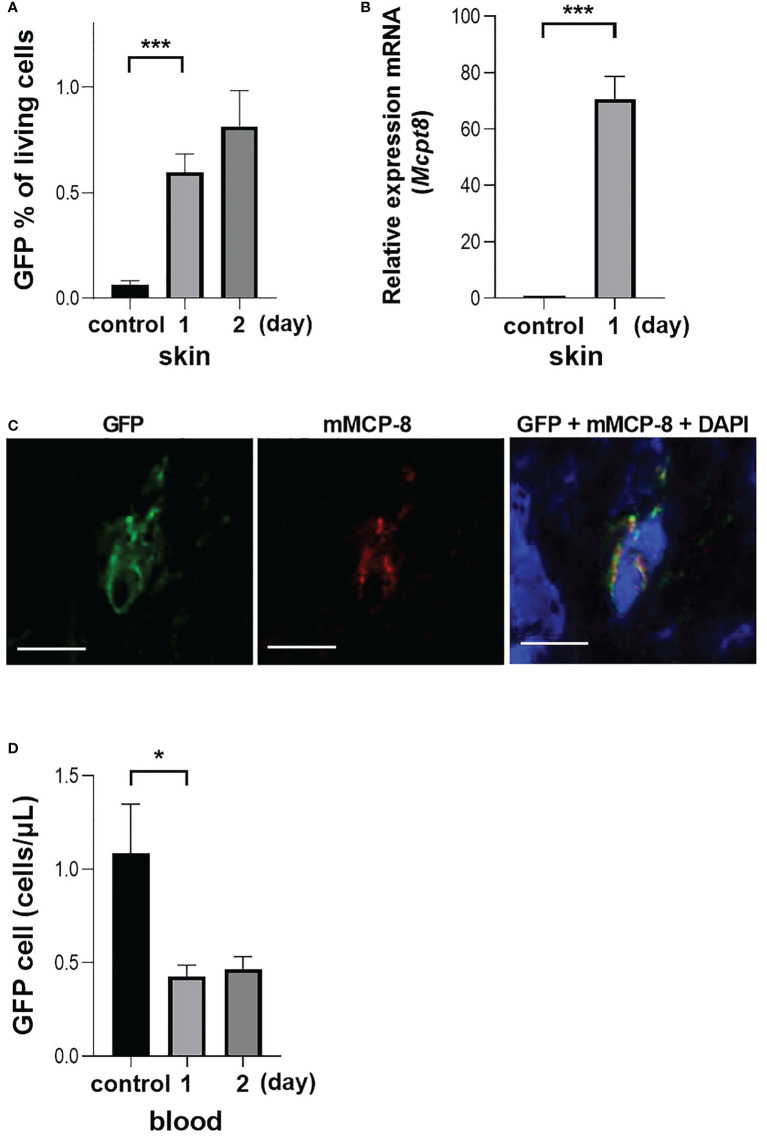
Basophil migration in an OX model with GFP-expressing BMBa transplantation. Each experiment was conducted at least 3 times individually (each group, n = 3), and data from one of these experiments are shown as representative. **(A)** GFP positive basophil count in skin at one and two days after challenge. **(B)** Q-PCR of *Mcpt8* in skin tissue. **(C)** Representative image of staining of skin tissue one day after challenge. Left panel: GFP-positive cells. Middle panel: anti-mMCP-8 staining with TUG8. Right panel: overlay with GFP, mMCP-8 and DAPI, scale bar = 5 µm. **(D)** GFP positive basophil count in blood at 1 and 2 days after challenge. Control mice were treated with BMBa but neither sensitized nor challenged, and samples were taken on day 2 after *i.v.* injection of BMBa. *p < 0.05 and ***p < 0.005.

Following BMBa transplantation, there were higher numbers of GFP-positive cells in skin tissue on the first day after challenge, but not on the second day (**Figure 4A**). When mRNA was extracted from skin tissue and examined using quantitative PCR, the expression of *Mcpt8* was higher in the tissue collected one day after challenge (**Figure 4B**). One day after the challenge, there was an infiltration of cells in the dermis for which the cytoplasm had granular GFP positive staining (**Figure 4C**). Finally, the number of GFP-expressing basophils in the PB was observed over time, and we can confirm that GFP-positive cells in the PB were lower after 1 day of challenge and there was no apparent increase after two days (**Figure 4D**), suggesting that the recovery of the number of basophils in the PB observed on the second day of challenge reflected the mobilization of new basophils from the BM.

## Discussion

The impetus for this study came from our experience with one CSU patient, who　had had severe CSU for 6 years and had persistently low, almost undetectable peripheral basophil counts for at least 1 year (26). When urticaria was improved by treatment with omalizumab, we noticed that his peripheral basophil count recovered. When the patient discontinued omalizumab treatment, the PB basophil counts again dropped to zero and urticaria recurred. Re-administration of omalizumab improved the skin rash and rescued the peripheral basophil count.

Our observation of an apparent basopenia associated with CSU is consistent with other reports (14–16). Of particular interest has been suggestions that a reduced basophil count may predict omalizumab efficacy (11, 15–18). Johal et al. (17) reported that those with decreased PB basophils had higher symptom scores and slower symptom improvement with omalizumab treatment than those without. Rijavec et al. (15) reported very low absolute basophil counts in circulating blood (1.7 basophils/μL) was reported to be a predictor of poor response to omalizumab. However, the mechanism of basopenia during the active phase of urticaria has not been clarified (27).

In a report examining various inflammatory diseases by immunostaining with basophil-specific antibody BB1, basophils were detected in 6 out of 10 cases of urticaria examined (20). In fact, in our CSU patients who had previously undergone skin biopsies, we found that while basophils were rarely seen in the skin of non-inflamed healthy controls, basophils were seen to varying degrees in 12 out of 22 CSU cases in urticarial lesions, as shown in **Table 1**. Based on these observations, we hypothesized that the decrease in basophils in the PB during the active phase of urticaria reflects the migration of basophils to the cutaneous region. However, skin biopsies are not usually performed for urticaria. In addition, many refractory cases that have undergone biopsy have already been treated with oral steroids at the time of skin biopsy. Therefore, it is difficult to correlate the migration of basophils to skin tissue with PB.

Although there is no suitable mouse model that reproduces the pathogenesis of urticaria, repeated application of OX has been reported to shift the immune response toward Th2 and induce migration of basophils to the lesion site (25). In this report, basophils were reported to infiltrate the skin even two days after a single application of OX. In addition, very interestingly, the latest single cell RNA sequencing analysis shows that basophils, which are newly migrated to the skin by OX treatment, are the source of IL-4 and IL-13 and tilt the immune response toward Th2, rather than mast cells residing in the tissues (28). In our present study, 5 days after sensitization, a single challenge of OX resulted in migration of a sufficient number of basophils to the skin, though there was not a concomitant decrease in basophils in the PB. However, as the number of basophils in BM increased two days after challenge, prompting us to consider the possibility that the lack of a decrease in basophils in the PB may be a consequence of new basophils being supplied from BM to the peripheral circulation.

In our investigation of the kinetics of basophil migration in tissue, PB and BM in the mouse OX model, the number of basophils migrating to the tissues one day after challenge was not as high as at two days after. However, at this time point the number of basophils in the PB was clearly decreased, while that in the BM had not yet increased. Since urticaria generally resolves within 24 hours, the reaction after 1 day of challenge in the mouse model may reproduce features of human urticaria. In CSU, however, the migration of basophils is not necessarily a transient phenomenon, since even after one skin rash disappears, another rash may appear at a different site. As a result, new basophils are recruited from the BM to the peripheral circulation to compensate for the shortage of basophils migrating to the skin, and the decrease of basophils in the PB should not be observed after the second day. However, the discrepancy observed in basophil kinetics between the mouse OX model and human urticaria may reflect differences between the mouse and human. In parasite infestation, basophilia in the PB has been observed in mice, whereas in humans, an increase in basophil numbers was reported to be exceedingly rare (29). In addition, in clinical practice, while neutrophilia or eosinophilia are often encountered, there are few diseases associated with increased basophil numbers other than rare basophilic leukemias. Thus, human BM production of basophils may be severely restricted. To exclude the influence of new basophils emerging from BM, in the present study BMBa expressing GFP were transferred into the PB and their contribution could be distinguished from that of cells mobilized from BM. As expected, we observed a decrease in the number of basophils in the PB, reflecting the migration of basophils to the inflamed skin, regardless of mobilization from BM.

In this study, we used a single application of OX to study the migration of basophils to the skin. Given the success of omalizumab targeting IgE in CSU, we may consideration should be given to a model in which there is cell migration or activation *via* IgE. In that case, however, the influence of not only basophils but also other IgE-mediated activated cells, especially mast cells pre-localized in the skin, would need to be considered.　In addition, it has been reported that mast cells are also required for contact hypersensitivity, since symptoms are reduced in mast cell-deficient mice (30–32). On the other hand, studies in models of atopic dermatitis with repeated applications of OX have shown that skin thickness is reduced, even in the absence of basophils (25). We believe that further investigation is needed to determine whether IgE-mediated stimulation also reduces the number of basophils in the PB, reflecting the migration of basophils to the skin area, and whether there is any interrelationship between the roles of basophils and mast cells.

The number of basophils in PB may be useful as an index of urticarial activity. Even though the studies we report here have the limitation that they are experiments with different species and models and that it is impossible to explain their results in a unified manner, we believe that one of the mechanisms by which the number of basophils in PB decreases at the onset of urticaria is that basophils migrate from the circulation into the skin. A focus on basophils should lead to new understanding of the pathogenesis of CSU.

## Data availability statement

The original contributions presented in the study are included in the article. Further inquiries can be directed to the corresponding author.

## Ethics statement

The studies involving human participants were reviewed and approved by Ethics Committee of Kansai Medical University. The patients/participants provided their written informed consent to participate in this study. The animal study was reviewed and approved by Animal Ethics Committee of Kansai Medical University

## Author contributions

IK, HT, and NK designed the experiments. NM, RT-I, CN, AO, and AW supported the planning and validated the strategy. IK, NK, RT-I, HT, and CN performed experiments. IK, NM, and NK wrote the manuscript. IK, NM, CN, HT, and NK performed data analysis. All authors contributed to the article and approved the submitted version.

## Funding

This work was supported by JSPS KAKENHI Grant Number 20K17364 (IK).

## Acknowledgments

We thank Drs. Kensuke Miyake and Hajime Karasuyama (Tokyo Medical and Dental University) for providing us with *Mcpt8^GFP^* mice and teaching us to identify basophils in the OX-based model; and Dr. Marcus Maurer (Charité - Universitätsmedizin Berlin) for the opportunity to discuss our project.

## Conflict of interest

The authors declare that the research was conducted in the absence of any commercial or financial relationships that could be construed as a potential conflict of interest.

## Publisher’s note

All claims expressed in this article are solely those of the authors and do not necessarily represent those of their affiliated organizations, or those of the publisher, the editors and the reviewers. Any product that may be evaluated in this article, or claim that may be made by its manufacturer, is not guaranteed or endorsed by the publisher.
